# The Association Between Variability in Electrolytes and the In-Hospital Mortality in Critically Ill Children in Pediatric Intensive Care Units

**DOI:** 10.3389/fped.2021.692894

**Published:** 2021-08-03

**Authors:** Jilei Lin, Yin Zhang, Meng Chen, Jihong Dai, Anchao Song, Jianchuan Chen, Xingping Tao

**Affiliations:** ^1^Department of Respiratory Medicine, Shanghai Children's Medical Center, Shanghai Jiaotong University School of Medicine, Shanghai, China; ^2^Department of Respiratory and Critical Care Medicine, West China Hospital of Sichuan University, Chengdu, China; ^3^Department of Pediatrics, Meitan People's Hospital, Zunyi, China; ^4^Chongqing Key Laboratory of Pediatrics, Ministry of Education Key Laboratory of Child Development and Disorders, Department of Respiratory Disease, National Clinical Research Center for Child Health and Disorders, Children's Hospital of Chongqing Medical University, Chongqing, China; ^5^School of Public Health and Management, Chongqing Medical University, Chongqing, China; ^6^Department of Pediatrics, Kaiyuan People's Hospital, Kaiyuan, China

**Keywords:** electrolytes, potassium, sodium, in-hospital mortality, variability, children

## Abstract

**Objective:** This study aimed to explore the association between the variability in electrolytes and the in-hospital mortality in critically ill children admitted into intensive care units (ICUs).

**Design:** This is a retrospective case–control study.

**Setting and Participants:** Total of 11,245 children have been admitted to ICUs of Children's Hospital of Zhejiang University from 2010 to 2018.

**Methods:** The coefficient of variation (CV), standard deviation (SD), and variability independent of the mean (VIM) were calculated as variability indices. High variability was defined as having values in the highest quartile for each parameter. Age, sex, diagnoses of disease, and surgical treatment were adjusted in the multivariable-adjusted logistic regression model.

**Results:** A total of 11,245 children were included, and 660 patients died in the hospital. The median (P25, P75) potassium, sodium, and chloride of all patients were 3.8 (3.58, 4.09), 136.83 (135.11, 138.60), and 108.67 (105.71, 111.17), respectively. U-shaped relationships between the mean, lowest, and highest levels of potassium, sodium, and chloride and the in-hospital mortality were observed. The lowest mortality was noted when serum potassium, sodium, and chloride were between ~3.5 and 5.0, 135 and 145, and 105 and 115 mmol/l, respectively. The areas under the curve (AUCs) of three indices of variability in electrolytes were larger than those of the mean and lowest levels of electrolytes in predicting the in-hospital mortality. In the multivariable-adjusted model, the odds ratios and 95% confidence interval (CI) of the in-hospital mortality were 3.14 (2.44–4.04) for one parameter, 5.85 (4.54–7.53) for two parameters, and 10.32 (7.81–13.64) for three parameters compared with subjects having no parameters of high variability measured as the CV. The results were consistent when the variability was determined using the SD and VIM (all *P* for trend <0.001). Consistent results were noted in various subgroup analyses.

**Conclusions:** This study showed that individuals with higher variability of each parameter were related with higher risk of in-hospital mortality. There was a linear association between the number of high variability parameters and the in-hospital mortality. The variability of electrolytes might be a good predictor for in-hospital mortality of children in ICUs.

## Introduction

An adequate electrolyte balance is of paramount importance to maintaining physiologic homeostasis and normal metabolism of cells in human body. Internal environment imbalance and electrolyte disorders usually occur in critically ill patients admitted to intensive care units (ICUs), due to different causes. Prompt recognition of electrolyte disorders is essential to the care and treatment of these patients (1, 2).

To our knowledge, blood gas analysis is conducted regularly and frequently in critically ill patients in ICUs to monitor patients' physiologic homeostasis. The main items of electrolytes in blood gas analysis are potassium, sodium, and chloride in ICUs. Sodium (Na^+^) and chloride (Cl^−^) are the major extracellular ions, while potassium (K^+^) is the major intracellular cation in the body, and they serve essential functions in the membrane potential and metabolism. ^1^ Unbalanced homeostasis will affect neural transmission, vascular tone, etc. and may even lead to life-threatening complications. Dyskalemia is known to induce potentially lethal arrhythmias and cardiac dysfunction, as well as other complications, even mortality risk (3–6). Dysnatremia is also a common finding in ICUs and has been suggested to be a predictor for poor clinical outcomes in patients with different dysfunctions (7–9). Besides, previous studies showed hypo- and hyperchloremia may contribute to higher mortality in patients with major trauma, chronic heart failure, and severe sepsis (10–12). However, it is inappropriate to evaluate the association between serum electrolytes and mortality at a screening visit or using the mean values for pediatric patients with critical illness during the hospitalization, since the serum levels of electrolytes may vary and the normal range of the values is wide in children with different ages. The value of electrolytes may change a lot by the treatment during the hospitalization, so it is inappropriate to use an extreme value to predict the mortality of critically ill children. Therefore, it is important to choose a better way to fit the relationship between electrolytes and in-hospital mortality in critically ill children, which can reduce the influence of baseline and extreme values.

Recently, some studies showed that the indices of variability [coefficient of variation (CV), standard deviation (SD) and variability independent of the mean (VIM)], which can reduce the influence of the baseline level of parameters, have emerged as previously unrecognized residual risk factors that is related to the development of various health outcomes (13–15). Therefore, this study aimed to evaluate the association between the variability in electrolytes (including potassium, sodium, and chloride) and in-hospital mortality in critically ill children admitted into ICUs, with a large-scale sample size.

## Methods

### Study Population

The data of this observational case–control study were extracted from the Pediatric Intensive Care (PIC) database, a freely accessible pediatric-specific critical care database established by the Children's Hospital of Zhejiang University, a 1,900-bed children's hospital in the south of China (16). In this study, patients have been all admitted to ICUs during a period of 8 years from 2010 to 2018. A total of 13,449 admissions were recorded in the PIC database, in which 971 (7.2%) patients died in hospital. Structured clinical data including the patient demographics, symptoms, vital signs, laboratory results, prescriptions, and surgeries were all collected in the database. The project was approved by the Institutional Review Board of the Children's Hospital, Zhejiang University School of Medicine (Hangzhou, China). The requirement for individual patient consent was waived because the project did not impact clinical care, and all protected health information was deidentified. We accessed the PIC database under a data use agreement from a database manager (Prof. Minhao Li). The results of this study were reported following the Strengthening the Reporting of Observational Studies in Epidemiology statement (17).

### Data Extraction

We included patients if they were admitted to ICUs for the first time and their values of three serum electrolytes (including potassium, sodium, and chloride) were recorded for no less than three times during this hospitalization. However, we excluded patients whose information was seriously absent or wrongly recorded. In this study, the collected data included age, gender, diagnoses of disease (including diseases of the respiratory system, nervous system, circulatory system, digestive system, genitourinary system, and neoplasms), whether surgery was performed or not in this hospital stay, and the length of ICU stay. Of note, the International Classification of Diseases-10 (ICD-10) code was used to classify diseases of different systems. The endpoint of this study was the in-hospital mortality.

### Definitions of Variability Indices

The in-hospital mortality referred to the rate of death in this hospital stay. Variability in each parameter (including potassium, sodium and, chloride) was defined as variability in their values. We used three indices of variability in this study: CV, SD, and VIM. To explain, the SD was calculated as sqrt{[(*x*_1_ – *x*)^∧^2 + (*x*_2_ – *x*)^∧^2 +. (*x*_*n*_ – *x*)^∧^2]/(*n* – 1)}; the CV was calculated as SD/mean; and the VIM was calculated as × SD/mean^β^, where β is the regression coefficient, based on the natural logarithm of the SD over the natural logarithm of the mean (18). Specially, each parameter was divided by quartiles, and high variability was defined as values in the highest quartile. The patients were classified further according to the number of high variability parameters using a score ranging from 0 to 3. In this classification, a score of 0 indicated no high variability parameter, and the scores 1 to 3 indicated the number of high variability parameters among the three serum electrolytes (e.g., a score of 2 indicated high variability in two of the three parameters).

### Statistical Analysis

In this study, continuous variables were presented as means ± SD. Categorical variables were analyzed by the χ^2^ test or the Fisher's exact test, as appropriate. They were expressed as numbers (*n*) with percentages (%). The area under the curve (AUC) of receiver operating characteristic (ROC) was used to evaluate the discrimination ability of the mean, lowest, and highest levels of electrolytes and the variability of them. In order to assess the relationship between the mean, lowest, and highest levels of potassium, sodium, and chloride and in-hospital mortality, line charts were calculated and presented in three indices of variability. To test whether a trend across quartiles of three indices of variability of potassium, sodium, and chloride existed for risk estimates and to explore the relationship between the number of parameters with high variability and the in-hospital mortality, logistic regression was used to calculate the odds ratios (ORs) and 95% confidence intervals (CIs). Age, sex, diagnoses of disease, and surgical treatment were adjusted in the multivariable-adjusted logistic regression model 1, while model 2 was further adjusted for mean levels of potassium, sodium, and chloride. Subgroup analyses were planned to be performed to evaluate whether the observed association of the number of parameters with high variability with the in-hospital mortality was modified by age, sex, or surgical treatment. *P* < 0.05 was considered to indicate statistical significance.

Data extraction was conducted using PostgreSQL. All statistical analyses were performed using Stata 15.1 software and R 3.61 software.

## Results

### Study Subject Characteristics

According to the eligibility criteria and exclusion criteria, a total of 11,245 children were included. The median (P25, P75) potassium, sodium, and chloride of all patients were 3.8 (3.58, 4.09), 136.83 (135.11, 138.60), and 108.67 (105.71, 111.17), respectively. The detailed mean values of these electrolytes in each group are shown in Table 1 and Supplementary Table 2. There were 5,916, 2,935, 1,681, and 713 children having 0, 1, 2, or 3 parameters with high variability measured by CV, respectively. Children's baseline characteristics by the number of parameters with high variability measured by CV are shown in Table 1. Patients who had more parameters with high variability had lower proportion of surgical experience. They also had higher variability in potassium, sodium, and chloride. Furthermore, these patients had longer length of PICU stay and higher in-hospital mortality. Similar subject characteristics were observed when the variability was calculated as the SD and VIM (see Supplementary Tables 1, 2).

**Table 1:** Characteristics of subjects according to number of parameters with high variability measured as coefficient of variation.

	**0 parameter** **(*n* = 5,916)**	**1 parameter** **(*n* = 2,935)**	**2 parameters** **(*n* = 1,681)**	**3 parameters** **(*n* = 713)**
**Demographic characteristics**				
Age (mean ± SD, years old)	2.86 ± 3.76	1.96 ± 3.28	2.03 ± 3.4	1.95 ± 3.33
Gender {male [*n* (%)]}	3,359 (56.78)	1,689 (57.55)	961 (57.17)	427 (59.89)
**Diagnoses of disease [** ***n*** **(%)]**				
Diseases of the respiratory system	554 (9.36)	300 (10.22)	147 (8.74)	69 (9.68)
Diseases of the nervous system	388 (6.56)	180 (6.13)	136 (8.09)	56 (7.85)
Diseases of the circulatory system	359 (6.07)	242 (8.25)	156 (9.28)	80 (11.22)
Diseases of the digestive system	566 (9.57)	215 (7.33)	85 (5.06)	32 (4.49)
Diseases of the genitourinary system	254 (4.29)	76 (2.59)	29 (1.73)	20 (2.81)
Diseases of the immune system	178 (3.01)	60 (2.04)	31 (1.84)	26 (3.65)
Neoplasms	291 (4.92)	81 (2.76)	72 (4.28)	31 (4.35)
**Surgical treatment [** ***n*** **(%)]**	3,440 (58.15)	1,373 (46.78)	699 (41.58)	232 (32.54)
**Potassium (mmol/l)**				
Mean	3.85 ± 0.37	3.89 ± 0.49	3.86 ± 0.55	3.89 ± 0.61
SD	0.38 ± 0.13	0.61 ± 0.4	0.67 ± 0.47	0.94 ± 0.58
VIM	7.95 ± 2.68	12.37 ± 5.82	13.75 ± 6.7	19.06 ± 8.92
CV	9.86 ± 3.32	15.38 ± 7.43	17.09 ± 8.57	23.71 ± 11.41
**Sodium (mmol/l)**				
Mean	136.88 ± 2.43	136.91 ± 3.46	137.25 ± 5.71	138.37 ± 8.35
SD	2.52 ± 0.89	3.65 ± 1.64	5.62 ± 2.61	7.52 ± 3.82
VIM	0 ± 0	0 ± 0	0 ± 0	0 ± 0
CV	1.84 ± 0.65	2.67 ± 1.19	4.08 ± 1.81	5.38 ± 2.49
**Chloride (mmol/l)**				
Mean	108.8 ± 3.59	108.16 ± 4.64	107.52 ± 6.43	107.59 ± 8.32
SD	2.91 ± 1.06	4.17 ± 1.94	6.12 ± 2.53	7.92 ± 3.19
VIM	2,7324.2 ± 9,956.72	38,955.26 ± 18,196.28	57,077.07 ± 25,318.18	74,377.23 ± 33,739.14
CV	2.68 ± 0.98	3.87 ± 1.81	5.7 ± 2.28	7.35 ± 2.82
**Length of ICU stay (days)**	7.22 ± 16.71	11.07 ± 18.66	14.04 ± 21.49	16.87 ± 23.07
**In-hospital death [** ***n*** **(%)]**	101 (1.71)	197 (6.71)	216 (12.85)	166 (23.28)

*Data were expressed as mean ± standard deviation or n (%)*.

*CV, coefficient of variation; SD, standard deviation; VIM, variability independent of the mean*.

### The Association Between Electrolytes and the In-Hospital Mortality

Line charts were conducted to assess the relationship between the mean, lowest, and highest levels of potassium, sodium, and chloride and the in-hospital mortality. To conclude, U-shaped relationships were observed. The lowest mortality was noted when serum potassium, sodium, and chloride was between ~3.5 and 5.0, 135 and 145, and 105 and 115 mmol/l, respectively (see Supplementary Figure 1).

The ROCs of the mean, lowest, and highest levels of electrolytes and the variability of them were plotted for in-hospital mortality (see Supplementary Figure 2). For potassium, although the highest levels of potassium had a good performance [OR (95% CI): 0.753 (0.732–0.774)], the three indices of variability showed larger AUCs [CV: 0.729 (95% CI 0.708–0.749), SD: 0.743 (95% CI 0.723–0.763), and VIM: 0.725 (95% CI 0.704–0.745)] than the mean [OR (95% CI): 0.595 (0.569–0.621)] and lowest [OR (95% CI): 0.610 (0.584–0.635)] levels of potassium in predicting the in-hospital mortality. For sodium, the three indices of variability showed larger AUCs [CV: 0.707 (95% CI 0.685–0.728), SD: 0.704 (95% CI 0.683–0.726), and VIM: 0.707 (95% CI 0.661–0.705)] than the mean [OR (95% CI): 0.515 (0.486–0.543)], lowest [OR (95% CI): 0.633 (0.608–0.658)], and highest [OR (95% CI): 0.652 (0.626–0.678)] levels of sodium in predicting the in-hospital mortality. For chloride, the three indices of variability showed larger AUCs [CV: 0.710 (95% CI 0.689–0.732), SD: 0.698 (95% CI 0.676–0.720), and VIM: 0.683 (95% CI 0.668–0.829)] than the mean [OR (95% CI): 0.638 (0.611–0.665)] and highest [OR (95% CI): 0.533 (505–0.561)] levels of chloride in predicting the in-hospital mortality, though the lowest levels of chloride showed a good performance [OR (95% CI): 0.721 (0.700–0.743)].

### Variability of Individual Parameters and the In-Hospital Mortality

An incrementally higher in-hospital mortality was observed with the highest CV quartile of potassium, sodium, and chloride compared to the lowest quartile groups, unadjusted [OR (95% CI): Q4 of K^+^, 13.39 (6.45–27.81); Q4 of Na^+^, 6.03 (3.66–9.94); Q4 of Cl^−^, 4.24 (2.71–6.64)]; adjusted for age, sex, different diagnoses of disease, and surgery treatment (model 1) [OR (95% CI): Q4 of K^+^, 11.18 (5.33–23.45); Q4 of Na^+^, 4.74 (2.84–7.91); Q4 of Cl^−^, 3.01 (1.89–4.80)]; and even further adjusted for mean levels of potassium, sodium, and chloride (model 2) [OR (95% CI): Q4 of K^+^, 10.12 (4.78–21.42); Q4 of Na^+^, 4.63 (2.72–7.89); Q4 of Cl^−^, 2.72 (1.66–4.45)] (Table 2). Similar findings were noted when the variability was determined using the SD and VIM (all *p* for trend <0.001) (see Supplementary Tables 3, 4).

**Table 2:** Odds ratios and 95% confidence intervals of in-hospital mortality by quartiles of electrolyte variability measured as coefficient of variation.

	**OR (95%CI)**		
	**Unadjusted**	Model 1^*^	Model 2^**^
**Potassium**			
Q1	1.00 (reference)	1.00 (reference)	1.00 (reference)
Q2	**3.85 (1.74–8.52)**	**3.84 (1.72–8.60)**	**3.78 (1.68–8.51)**
Q3	**6.02 (2.79–13.00)**	**5.12 (2.35–11.15)**	**4.49 (2.04–9.87)**
Q4	**13.39 (6.45–27.81)**	**11.18 (5.33–23.45)**	**10.12 (4.78–21.42)**
*P* for trend	<0.001	<0.001	<0.001
**Sodium**			
Q1	1.00 (reference)	1.00 (reference)	1.00 (reference)
Q2	1.64 (0.88–2.91)	1.44 (0.78–2.65)	1.53 (0.82–2.88)
Q3	**3.19 (1.85–5.49)**	**2.60 (1.49–4.53)**	**2.39 (1.34–4.24)**
Q4	**6.03 (3.66–9.94)**	**4.74 (2.84–7.91)**	**4.63 (2.72–7.89)**
*P* for trend	<0.001	<0.001	<0.001
**Chloride**			
Q1	1.00 (reference)	1.00 (reference)	1.00 (reference)
Q2	1.11 (0.64–1.91)	0.95 (0.54–1.66)	0.91 (0.51–1.63)
Q3	**1.86 (1.12–3.10)**	1.49 (0.88–2.52)	1.37 (0.79–2.38)
Q4	**4.24 (2.71–6.64)**	**3.01 (1.89–4.80)**	**2.72 (1.66–4.45)**
*P* for trend	<0.001	<0.001	<0.001

* *Model 1 was adjusted for age, sex, diagnoses of disease, and surgical treatment*.

** *Model 2 was adjusted for model 1 plus mean values of electrolytes (potassium, sodium, and chloride)*.

*Bold data are data with statistical significance*.

### Number of Parameters With High Variability and the In-Hospital Mortality

Furthermore, the composite effect of parameter variability on the in-hospital mortality was evaluated. An incrementally higher in-hospital mortality was noted with a higher number of parameters with high variability (Table 3). After adjusting for possible confounding factors mentioned before, the ORs (95% CI) of in-hospital mortality were 3.14 (2.44–4.04) for one parameter, 5.85 (4.54–7.53) for two parameters, and 10.32 (7.81–13.64) for three parameters compared with children having no parameters of high variability measured as the CV. The results were consistent when the variability was determined using the SD and VIM (all *P* for trend <0.001).

**Table 3:** Odds ratios and 95% confidence intervals of in-hospital mortality by number of electrolytes with high variability.

**Number of electrolytes with high variability**	**OR (95%CI)**		
	**Unadjusted**	Model 1^*^	Model 2^**^
**Measured as CV**			
0	1.00 (reference)	1.00 (reference)	1.00 (reference)
1	**4.14 (3.25–5.29)**	**3.50 (2.73–4.48)**	**3.14 (2.44–4.04)**
2	**8.49 (6.66–10.83)**	**6.83 (5.33–8.76)**	**5.85 (4.54–7.53)**
3	**17.47 (13.44–22.72)**	**13.05 (9.96–17.09)**	**10.32 (7.81–13.64)**
*P* for trend	<0.001	<0.001	<0.001
**Measured as SD**			
0	1.00 (reference)	1.00 (reference)	1.00 (reference)
1	**3.83 (3.01–4.87)**	**3.24 (2.53–4.14)**	**2.87 (2.24–3.68)**
2	**8.17 (6.44–10.36)**	**6.60 (5.17–8.43)**	**5.44 (4.24–6.97)**
3	**16.66 (12.77–21.72)**	**11.65 (8.86–15.32)**	**8.83 (6.65–11.71)**
*P* for trend	<0.001	<0.001	<0.001
**Measured as VIM**			
0	1.00 (reference)	1.00 (reference)	1.00 (reference)
1	**3.72 (2.94–4.71)**	**3.14 (2.47–4.00)**	**2.93 (2.30–3.74)**
2	**7.32 (5.78–9.27)**	**5.98 (4.70–7.61)**	**5.50 (4.30–7.03)**
3	**15.18 (11.73–19.64)**	**11.45 (8.78–14.94)**	**9.63 (7.32–12.68)**
*P* for trend	<0.001	<0.001	<0.001

* *Model 1 was adjusted for age, sex, diagnoses of disease, and surgical treatment*.

** *Model 2 was adjusted for model 1 plus mean values of electrolytes (potassium, sodium, and chloride)*.

*CV, coefficient of variation; SD, standard deviation; VIM, variability independent of the mean*.

*Bold data are data with statistical significance*.

**Figure 1:**
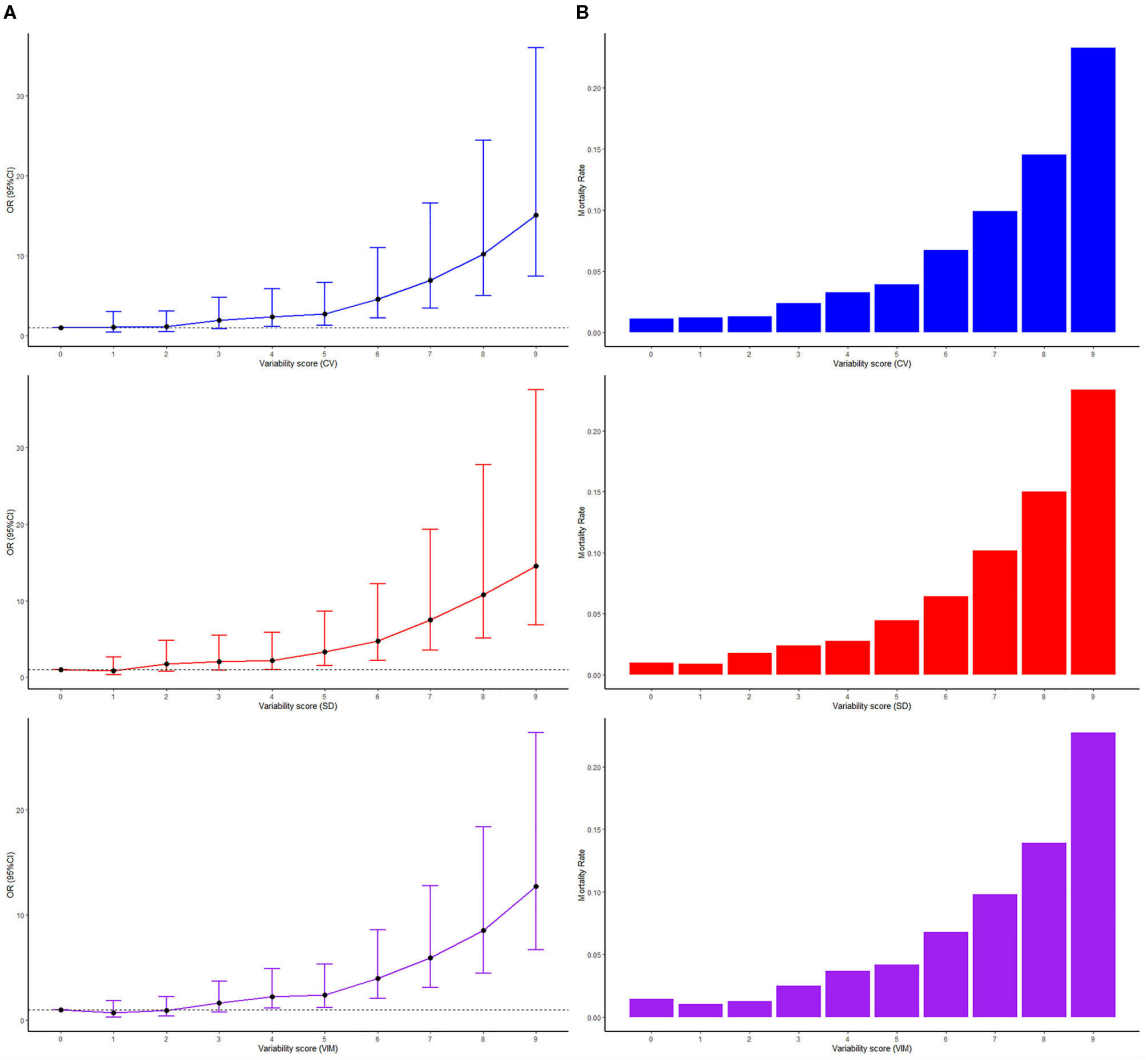
A positive linear association between the variability score and **(A)** odds ratios and 95% confidence interval for the in-hospital mortality (line charts) and **(B)** the in-hospital mortality (bar charts).

To evaluate the influence of lower quartile groups together, a variability scoring system was defined. In the system, 0 point was assigned for Q1, 1 point for Q2, 2 points for Q3, and 3 points for Q4 groups for each of three parameters. Thus, the total score ranged from 0 to 9. It was shown that there is a positive linear association between the variability score and the in-hospital mortality or OR for the in-hospital mortality (all *P* for trend <0.001) (Figure 1).

### Subgroup Analysis

**Figure 2:**
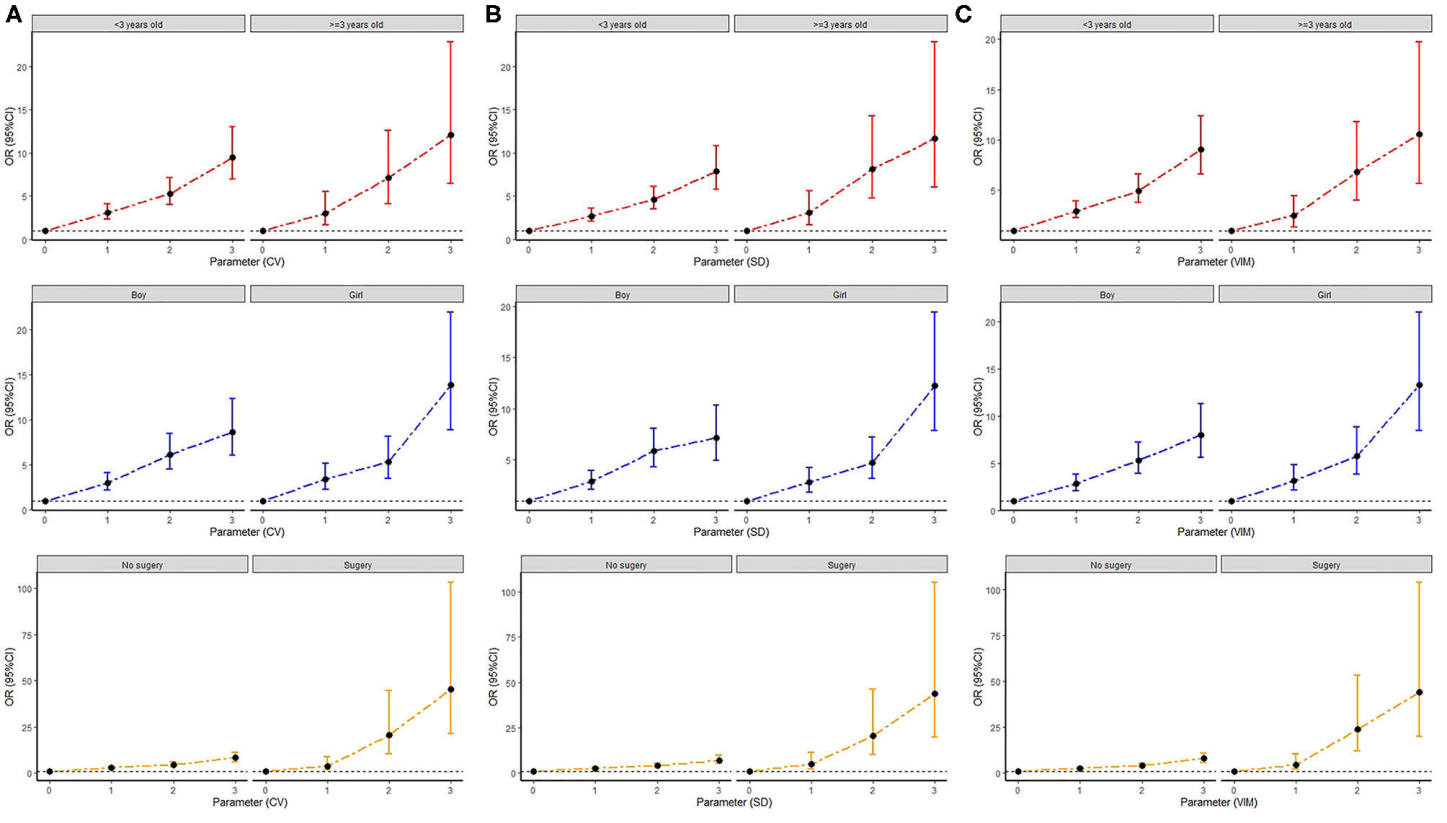
The odds ratios and 95% confidence interval for in-hospital mortality by number of parameters with high variability defined as the highest quartile of **(A)** CV of electrolytes; **(B)** SD of electrolytes; and **(C)** VIM of electrolytes. Subgroup analyses according to age, sex, and surgical experience. CV, coefficient of variation; SD, standard deviation; VIM, variability independent of the mean.

Pre-specified stratified analyses by age, sex, and surgical treatment were conducted. The significant association between the number of parameters with high variability and the in-hospital mortality was presented in all subgroups (Figure 2). Higher adjusted ORs for the in-hospital mortality were observed in surgical patients (all *p* for interaction <0.001), when the variability was determined using all of the three indices of variability. In addition, female subgroups showed higher in-hospital mortality when the variability was determined using the SD (*p* for interaction = 0.038). However, other subgroups showed similar in-hospital mortality with high variability.

## Discussion

This study showed a U-shaped association between the level of serum potassium, sodium, and chloride and the in-hospital mortality of children in ICUs. We also examined the association between three indices of variability of these electrolytes and the in-hospital mortality. Individuals with higher variability of each parameter were at higher risk of in-hospital mortality. The results were consistent in different subgroups, promising that the relationship was widely applicable. Of note, the result of ROC showed that the variability was more sensitive and stable than the mean, lowest, and highest level of these parameters as risk factors of the in-hospital mortality in critically ill children admitted in ICUs.

In clinical practice, normal ranges of levels of serum potassium, sodium, and chloride are established mainly based on healthy population, and it is still unclear whether these values are also applicable to children because there has been few evidence for clear recommendations on electrolyte management in critically ill children. We observed that children who had electrolytes in approximately normal ranges still had lower in-hospital mortality. The lowest mortality was noted when serum potassium, sodium, and chloride was between 3.5 and 5.0, 135 and 145, and 105 and 115 mmol/l, respectively. However, the level of serum chloride in correlation with the lowest mortality differed from the normal range between 95 and 100 mmol/l, indicating that the normal range of the electrolytes may not fit to all critically ill children. It is hard to make a regular summary for different levels of these parameters.

Recently, the variability in serum electrolytes has gained much attention as novel risk factors for in-hospital mortality in critically ill patients (19–21). Previous data suggested that the variability of potassium and sodium induced or reflected pathological conditions associated with mortality of critically ill patients, for it was related to membrane potential. Our results showed that the composite effect of potassium, sodium, and chloride variability was strongly related with the in-hospital mortality. Thus, the importance of stabilizing the variability of potassium, sodium, and chloride should be further emphasized. In this study, we observed that the variability of potassium, sodium, and chloride was more sensitive and stable in predicting the in-hospital mortality of critically ill children than the absolute levels of these parameters. Moreover, there was a positive linear relationship between the variability and the in-hospital mortality, which is more applicable in the clinical practice, compared to the level of these parameters. Considering that age and gender might affect the levels of electrolytes, and some studies showed that the imbalance in potassium and sodium may be related with early mortality following surgery (22, 23), we conducted the subgroup analyses to explore the relationship between variability indices and the in-hospital mortality in specific populations. As a result, higher adjusted ORs for in-hospital mortality were observed in the surgical group, which was consistent with previous studies (22, 23).

Critically ill children might have dysfunctions in organs that regulate electrolytes, such as the kidney, with consequent instability in several biological parameters, increasing the possibility of reverse causation. However, our data showed a strong correlation between variability indices and the in-hospital mortality with a dose–response relationship after adjusting potential confounding factors including the diagnosis of renal disease. The result was consistent with previous data, suggesting the electrolyte disturbance can directly increase the risk of mortality (24, 25). We infer that the variability in electrolytes is not only an indicator but also a risk factor for increased risk of severe health outcomes. We also performed subgroup analyses to strengthen the temporal relationship of the association and confirmed similar results. Our data suggested that the variability of these parameters is significantly related with the mortality, even in young children and other specific group population.

There were several strengths in this study. This is the first study, to our knowledge, exploring the association between different kinds of variability indices and mortality and compare the advantages and disadvantages between variability indices and the original values of electrolytes in critically ill children. The sample size of the present study was huge, allowing a relatively higher power to obtain the independent and joint prognostic effects of variability of serum potassium, sodium, and chloride. The most important point is that variability indices could reduce the bias caused by the treatment during the hospitalization, such as supplementation of potassium, sodium, and chloride.

There are limitations that should be noted. First, it was a retrospective analysis, and there might be discrepancies between the actual mortality and the information recorded in the database. Second, this study was a single-center study, and we could only be able to establish an association, rather than a causal relationship. Third, the different examining time points and possible inconsistency with different ways of measuring within the study period may influence the variability indices. Furthermore, it is possible that some of the unknown factors, such as genetic factors, may influence the mortality, although we tried to adjust covariates to minimize this possibility.

## Conclusions

In summary, the effect of variability indices on predicting the in-hospital mortality in critically ill children in ICUs was observed in this study. There was a linear association between the number of high variability parameters and the in-hospital mortality. However, larger-scale prospective studies are needed to validate the association.

## Data Availability Statement

Publicly available datasets were analyzed in this study. This data can be found here: https://www.nature.com/articles/s41597-020-0355-4.

## Ethics Statement

The studies involving human participants were reviewed and approved by Institutional Review Board of the Children's Hospital, Zhejiang University School of Medicine (Hangzhou, China). Written informed consent from the participants' legal guardian/next of kin was not required to participate in this study in accordance with the national legislation and the institutional requirements.

## Author Contributions

JL conceptualized and designed the study, supervised data collection, carried out the initial analyses, and drafted the initial manuscript. JC and XT designed the data collection instruments and collected data. AS coordinated and supervised data collection, assisted in the statistical analysis, and carried out the initial analyses. JD and MC coordinated and supervised data collection and critically reviewed the manuscript for important intellectual content. YZ conceptualized and designed the study, supervised data collection, and reviewed and revised the manuscript. All authors have read and approved the final manuscript.

## Conflict of Interest

The authors declare that the research was conducted in the absence of any commercial or financial relationships that could be construed as a potential conflict of interest.

## Publisher's Note

All claims expressed in this article are solely those of the authors and do not necessarily represent those of their affiliated organizations, or those of the publisher, the editors and the reviewers. Any product that may be evaluated in this article, or claim that may be made by its manufacturer, is not guaranteed or endorsed by the publisher.

## Abbreviations

Na^+^: sodium
Cl^−^: chloride
K^+^: potassium
AUC: area under the curve
CIs: confidence intervals
CV: coefficient of variation
ICD-10: International Classification of Diseases-10
ICUs: intensive care units
ORs: odds ratios
ROC: receiver operating characteristic
SD: standard deviation
VIM: variability independent of the mean.
