# Supplementary material for: The Association Between Variability in Electrolytes and the In-Hospital Mortality in Critically Ill Children in Pediatric Intensive Care Units
Source: Front Pediatr. 2021 Aug 3;9:692894. doi: 10.3389/fped.2021.692894 (PMC8368981; doi:10.3389/fped.2021.692894)
Supplement: Supplementary file 1 [file Data_Sheet_1.PDF]

Supplementary Table 1. Characteristics of subjects according to number of parameters with high variability measured as standard deviation

	0 parameter	1 parameter	2 parameters	3 parameters
**Demographic characteristics**				
Age(Mean±sd, years old)	2.94 ± 3.8	1.87 ± 3.2	2 ± 3.35	1.79 ± 3.28
Gender[Male(n(%))]	3327 (56.99)	1735 (57.37)	975 (56.13)	399 (61.76)
**Diagnoses of disease[n(%)]**				
Diseases of the respiratory system	543 (9.3)	314 (10.38)	137 (7.89)	76 (11.76)
Diseases of the nervous system	386 (6.61)	176 (5.82)	143 (8.23)	55 (8.51)
Diseases of the circulatory system	357 (6.12)	250 (8.27)	163 (9.38)	67 (10.37)
Diseases of the digestive system	563 (9.64)	230 (7.61)	78 (4.49)	27 (4.18)
Diseases of the genitourinary system	254 (4.35)	76 (2.51)	28 (1.61)	21 (3.25)
Diseases of the immune system	173 (2.96)	65 (2.15)	33 (1.9)	24 (3.72)
Neoplasms	295 (5.05)	85 (2.81)	68 (3.91)	27 (4.18)
**Surgical treatment[n(%)]**	3409 (58.39)	1429 (47.26)	726 (41.8)	180 (27.86)
**Potassium(mmol/L)**				
Mean	3.82 ± 0.35	3.91 ± 0.49	3.87 ± 0.57	4.01 ± 0.6
SD	0.37 ± 0.13	0.61 ± 0.4	0.67 ± 0.46	0.97 ± 0.58
VIM	7.96 ± 2.77	12.34 ± 5.81	13.74 ± 6.61	19.23 ± 9.2
CV	9.85 ± 3.39	15.36 ± 7.43	17.08 ± 8.44	24.01 ± 11.7
**Sodium(mmol/L)**				
Mean	136.85 ± 2.44	136.82 ± 3.34	137.52 ± 5.94	138.46 ± 8.28
SD	2.52 ± 0.89	3.63 ± 1.66	5.66 ± 2.69	7.57 ± 3.8
VIM	0 ± 0	0 ± 0	0 ± 0	0 ± 0
CV	1.84 ± 0.65	2.66 ± 1.22	4.1 ± 1.85	5.42 ± 2.47
**Chlorine(mmol/L)**				
Mean	108.72 ± 3.64	108.09 ± 4.59	107.78 ± 6.49	108.03 ± 8.36
SD	2.9 ± 1.05	4.13 ± 1.9	6.21 ± 2.54	8.01 ± 3.24
VIM	27248.81 ± 9863.35	38497.28 ± 17823.6	57997.54 ± 25441.99	75425.89 ± 34111.8
CV	2.68 ± 0.99	3.83 ± 1.77	5.76 ± 2.29	7.41 ± 2.89
**Length of ICU stay(day)**	7.1 ± 16.54	11.02 ± 18.37	14.13 ± 22.08	17.79 ± 23.44
**In-hospital death[n(%)]**	105 (1.8)	198 (6.55)	226 (13.01)	151 (23.37)

Data were expressed as mean ± standard deviation or n (%)

CV coefficient of variation, SD standard deviation, VIM variability independent of the mean

Supplementary Table 2. Characteristics of subjects according to number of parameters with high variability measured as variability independent of the mean

	0 parameter	1 parameter	2 parameters	3 parameters
**Demographic characteristics**				
Age(Mean±sd, years old)	2.86 ± 3.77	1.95 ± 3.27	2.02 ± 3.37	1.99 ± 3.41
Gender[Male(n(%))]	3371 (57.24)	1689 (56.85)	964 (57.04)	412 (59.28)
**Diagnoses of disease[n(%)]**				
Diseases of the respiratory system	562 (9.54)	300 (10.1)	142 (8.4)	66 (9.5)
Diseases of the nervous system	388 (6.59)	175 (5.89)	141 (8.34)	56 (8.06)
Diseases of the circulatory system	360 (6.11)	252 (8.48)	150 (8.88)	75 (10.79)
Diseases of the digestive system	551 (9.36)	230 (7.74)	87 (5.15)	30 (4.32)
Diseases of the genitourinary system	248 (4.21)	81 (2.73)	30 (1.78)	20 (2.88)
Diseases of the immune system	173 (2.94)	63 (2.12)	33 (1.95)	26 (3.74)
Neoplasms	291 (4.94)	87 (2.93)	68 (4.02)	29 (4.17)
**Surgical treatment[n(%)]**	3411 (57.92)	1385 (46.62)	719 (42.54)	229 (32.95)
**Potassium(mmol/L)**				
Mean	3.85 ± 0.37	3.88 ± 0.49	3.85 ± 0.54	3.87 ± 0.61
SD	0.38 ± 0.14	0.6 ± 0.4	0.68 ± 0.48	0.93 ± 0.58
VIM	7.95 ± 2.68	12.27 ± 5.79	13.95 ± 6.86	18.98 ± 8.82
CV	9.87 ± 3.32	15.25 ± 7.39	17.34 ± 8.77	23.61 ± 11.27
**Sodium(mmol/L)**				
Mean	136.91 ± 2.4	136.9 ± 3.67	137.16 ± 5.57	138.41 ± 8.35
SD	2.52 ± 0.9	3.65 ± 1.66	5.6 ± 2.59	7.57 ± 3.85
VIM	0 ± 0	0 ± 0	0 ± 0	0 ± 0
CV	1.84 ± 0.65	2.67 ± 1.23	4.06 ± 1.79	5.41 ± 2.49
**Chlorine(mmol/L)**				
Mean	108.65 ± 3.64	108.23 ± 4.74	107.76 ± 6.35	107.99 ± 8.21
SD	2.89 ± 1.05	4.19 ± 1.89	6.1 ± 2.53	8.03 ± 3.23
VIM	27131.73 ± 9738.63	39159.49 ± 17916.96	56987.88 ± 25087.41	75582.8 ± 34206.33
CV	2.67 ± 0.99	3.88 ± 1.76	5.67 ± 2.3	7.43 ± 2.87
**Length of ICU stay(day)**	7.18 ± 16.58	11.34 ± 19.94	13.87 ± 20	16.41 ± 22.69

**In-hospital death[n(%)]**	112 (1.9)	200 (6.73)	210 (12.43)	158 (22.73)

Data were expressed as mean ± standard deviation or n (%)

CV coefficient of variation, SD standard deviation, VIM variability independent of the mean

Supplementary Table 3. Odds ratios and 95% confidence intervals of in-hospital mortality by quartiles of electrolyte variability measured as standard deviation

	OR (95%CI)		
	**Unadjusted**	**Model 1***	**Model 2****
**Potassium**			
Q1	1.00(reference)	1.00(reference)	1.00(reference)
Q2	1.42(0.99-2.03)	1.33(0.93-1.92)	1.26(0.88-1.82)
Q3	**3.33(2.43-4.57)**	**3.02(2.18-4.16)**	**2.73(1.97-3.77)**
Q4	**8.52(6.35-11.44)**	**6.57(4.85-8.91)**	**5.21(3.82-7.09)**
P for trend	<0.001	<0.001	<0.001
**Sodium**			
Q1	1.00(reference)	1.00(reference)	1.00(reference)
Q2	1.20(0.87-1.64)	1.15(0.83-1.58)	1.20(0.86-1.66)
Q3	**2.26(1.71-3.00)**	**2.19(1.64-2.91)**	**2.12(1.58-2.84)**
Q4	**5.51(4.26-7.12)**	**4.60(3.54-5.99)**	**4.09(3.12-5.37)**
P for trend	<0.001	<0.001	<0.001
**Chlorine**			
Q1	1.00(reference)	1.00(reference)	1.00(reference)
Q2	1.32(0.97-1.81)	1.25(0.91-1.71)	1.23(0.89-1.69)
Q3	**2.36(1.78-3.14)**	**2.22(1.66-2.96)**	**2.09(1.55-2.81)**
Q4	**5.56(4.28-7.21)**	**4.60(3.53-6.00)**	**4.04(3.07-5.32)**
P for trend	<0.001	<0.001	<0.001

*Model 1, adjusted for age, sex, diagnoses of disease and surgical treatment

**Model 2, adjusted for model 1 plus mean values of electrolytes (potassium, sodium and chlorine)

Bold data are data with statistical significance

Supplementary Table 4. Odds ratios and 95% confidence intervals of in-hospital mortality by quartiles of electrolyte variability measured as variability independent of the mean

	OR(95%CI)		
	**Unadjusted**	**Model 1***	**Model 2****
**Potassium**			
Q1	1.00(reference)	1.00(reference)	1.00(reference)
Q2	**1.46(1.05-2.04)**	**1.38(0.988-1.94)**	**1.34(0.95-1.88)**
Q3	**2.46(1.81-3.34)**	**2.32(1.70-3.17)**	**2.18(1.59-2.98)**
Q4	**7.38(5.60-9.74)**	**6.10(4.59-8.10)**	**5.34(4.00-7.12)**
P for trend	<0.001	<0.001	<0.001
**Sodium**			
Q1	1.00(reference)	1.00(reference)	1.00(reference)
Q2	1.18(0.85-1.63)	1.12(0.81-1.55)	1.20(0.86-1.68)
Q3	**2.41(1.81-3.21)**	**2.34(1.75-3.12)**	**2.40(1.79-3.24)**
Q4	**5.84(4.50-7.59)**	**4.74(3.63-6.18)**	**4.55(3.46-5.99)**
P for trend	<0.001	<0.001	<0.001
**Chlorine**			
Q1	1.00(reference)	1.00(reference)	1.00(reference)
Q2	1.35(0.99-1.82)	1.30(0.95-1.77)	1.28(0.93-1.75)
Q3	**2.29(1.73-3.02)**	**2.21(1.67-2.93)**	**2.13(1.59-2.85)**
Q4	**5.00(3.87-6.46)**	**4.31(3.32-5.59)**	**4.00(3.05-5.24)**
P for trend	<0.001	<0.001	<0.001

*Model 1, adjusted for age, sex, diagnoses of disease and surgical treatment

**Model 2, adjusted for model 1 plus mean values of electrolytes(potassium, sodium and chlorine)

Bold data are data with statistical significance

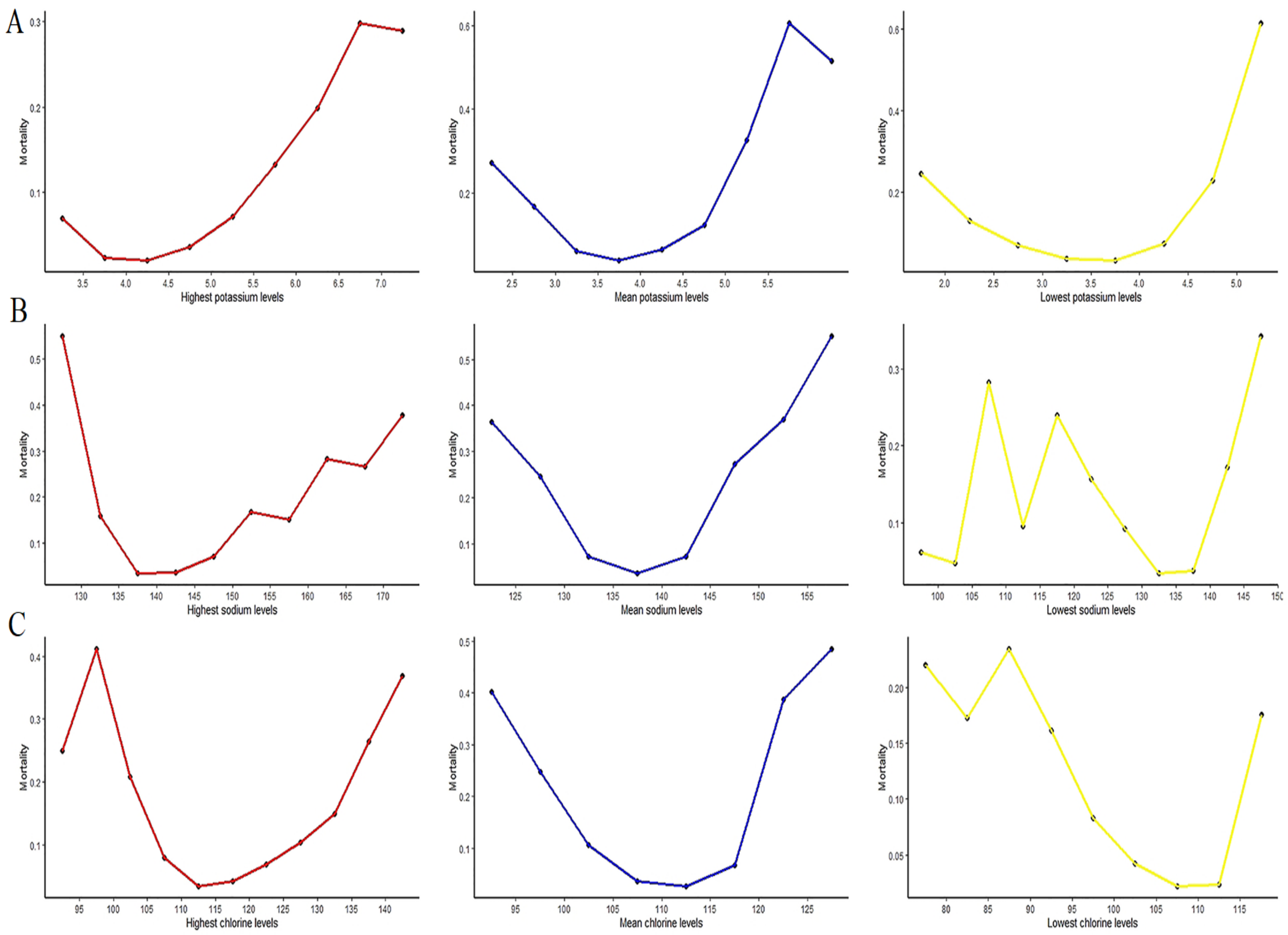

Supplementary Figure 1: Mean, lowest and highest serum potassium, sodium and chlorine levels and in-hospital mortality. Red line: highest level of electrolytes; Blue line: mean level of electrolytes; Yellow line: lowest level of electrolytes. A: serum potassium; B: serum sodium; C: serum chlorine.

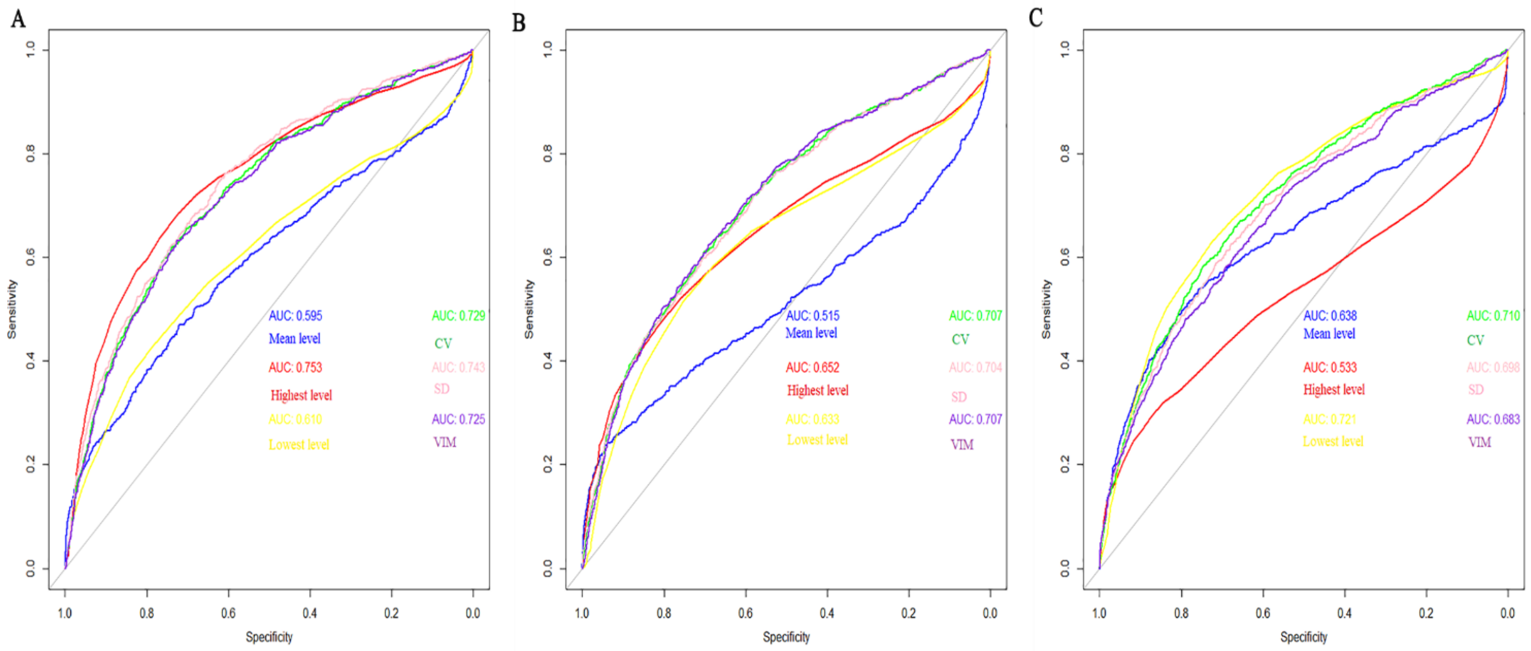

Supplementary Figure 2: ROCs of the serum potassium, sodium and chlorine in all included children. Red line: highest level of electrolytes; Blue line: mean level of electrolytes; Yellow line: lowest level of electrolytes; Green line: CV of electrolytes; Pink line: SD of electrolytes; Purple line: VIM of electrolytes. A: serum potassium; B: serum sodium; C: serum chlorine.
